# Design of Primers for Evaluation of Lactic Acid Bacteria Populations in Complex Biological Samples

**DOI:** 10.3389/fmicb.2018.02045

**Published:** 2018-08-31

**Authors:** Qiangchuan Hou, Xiaoye Bai, Weicheng Li, Xu Gao, Faming Zhang, Zhihong Sun, Heping Zhang

**Affiliations:** ^1^Key Laboratory of Dairy Biotechnology and Engineering, Ministry of Education, Inner Mongolia Agricultural University, Hohhot, China; ^2^Key Laboratory of Dairy Products Processing, Ministry of Agriculture, Inner Mongolia Agricultural University, Hohhot, China; ^3^Medical Center for Digestive Diseases, The Second Affiliated Hospital of Nanjing Medical University, Nanjing, China; ^4^Key Lab of Holistic Integrative Enterology, Nanjing Medical University, Nanjing, China

**Keywords:** lactic acid bacteria, microbial diversity, specific sequence primers, single molecule real-time sequencing, amplification

## Abstract

Lactic acid bacteria (LAB) are important for human health. However, the relative abundance of LAB in complex samples, such as fecal samples, is low and their presence and diversity (at the species level) is understudied. Therefore, we designed LAB-specific primer pairs based on 16S rRNA gene consensus sequences from 443 species of LAB from seven genera. The LAB strains selected were genetically similar and known to play a role in human health. Prior to primer design, we obtained consistent sequences for the primer-binding sites by comparing the 16S rRNA gene sequences, manually identifying single-stranded primers and modifying these primers using degenerate bases. We assembled primer pairs with product sizes of >400 bp. Optimal LAB-specific primers were screened using three methods: PCR amplification, agarose gel electrophoresis and single-molecule real-time (SMRT) sequencing analysis. During the SMRT analysis procedure, we focused on sequence reads and diversity at the species level of target LAB in three fecal samples, using the universal bacterium primer 27f/1492r as a reference control. We created a phylogenetic tree to confirm the ability of the best candidate primer pair to differentiate amongst species. The results revealed that LAB-specific primer L5, with a product size of 750 bp, could generate 3222, 2552, and 3405 sequence reads from fecal Samples 1, 2, and 3. This represented 14, 13 and 10% of all target LAB sequence reads, respectively, compared with 2, 0.8, and 0.8% using the 27f/1492r primer. In addition, L5 detected LAB that were in low abundance and could not be detected using the 27f/1492r primer. The phylogenetic tree based on the alignments between the forward and reverse primer of L5 showed that species within the seven target LAB genera could be distinguished from each other, confirming L5 is a powerful tool for inferring phylogenetic relationships amongst LAB species. In conclusion, L5 is a LAB-specific primer that can be used for high-throughput sequencing and identification of taxa to the species level, especially in complex samples with relatively low LAB content. This enables further research on LAB population diversity in complex ecosystem, and on relationships between LAB and their hosts.

## Introduction

Lactic acid bacteria (LAB) are a group of non-spore-forming, gram-positive bacteria that produce lactic acid as the main product of sugar fermentation (Zhang and Zhihong, 2015). LAB are the most common probiotics found in the human gut microbiota and in a range of fermented foods. Members of the genera *Lactococcus* and *Lactobacillus* have been accorded the status of “generally recognized as safe” (GRAS) (Salminen et al., 1998). LAB can prevent the adhesion and reproduction of pathogens in intestinal mucosal tissues, relieve the symptoms of intestinal problems, and release various enzymes into the gut that aid host digestion (Perez et al., 2014). Furthermore, LAB helps maintain the stability of intestinal microbial communities by producing extracellular polysaccharides that can be used by other intestinal microorganisms (Hidalgo-Cantabrana et al., 2014). Since different types of LAB can affect the human intestinal microenvironment in different ways, it is important to identify which microorganisms are present in a microbial ecosystem, and which species are most likely to have beneficial effects. Despite this, accurate identification of these bacteria at the species level is challenging.

In recent years molecular techniques have been developed that replace or complement traditional phenotypic methods of identification (Mohania et al., 2008). The most common technologies used include microarrays (Bae et al., 2005) and polymerase chain reaction (PCR)-based methods, such as Real-Time PCR (Furet et al., 2004) and PCR-DGGE (Muyzer et al., 1993; Hertel and Meroth, 2003). These methods are considered important for the detection and specific characterization of LAB. However, these methods are poor for observing and measuring the diversity of natural bacterial communities. Microarrays and Real-Time PCR require a predefined list of which species are being sought, and this generally comes from clone-based analyses. Missing out taxa at this early step can lead to their exclusion from subsequent, more-quantitative studies. PCR-DGGE also has much lower resolution than sequencing.

The development of molecular techniques based on sequence variability in the 16S rRNA genes has led to an improved understanding of the microbial communities present in a variety of ecosystems, including the gut microbiota (Suau et al., 1999; Satokari et al., 2003). Primer 27f/1492r is the most widely used primer for species-level identification (Frank et al., 2008). Currently available primers can reveal the composition of the predominant bacteria in a sample through amplification and sequencing. However, the average relative abundance of all LAB in the gut microbiota of healthy adults is very low, accounting for only 0.01∼1.8% of the total intestinal flora (Louis et al., 2007). Therefore, it is difficult to accurately reveal the composition of LAB in samples using only bacterial 16S universal primers. Some researchers have designed specific primers that amplify particular genera or species of LAB (Kao et al., 2007). However, these primers can also be limited. Either they only distinguish between species within the same LAB genus, or they only distinguish LAB at the genus level (Nakagawa et al., 1994; Walter et al., 2001; Heilig et al., 2002; Lopez et al., 2003; Moura et al., 2007; Delroisse et al., 2008). Therefore, in this study, we aimed to design a species-specific LAB primer pair to enable studies of the most common LAB populations in gut and fermented foods at species level. This includes species within the genera: *Lactobacillus*, *Streptococcus*, *Weissella*, *Lactococcus*, *Pediococcus*, *Enterococcus*, and *Leuconostoc*. This provides a valuable foundation for further studies on LAB populations.

## Materials and Methods

### Ethics Statement

The study protocol was approved by the Ethical Committee of the Inner Mongolia Agricultural University (Hohhot, China) and the Second Affiliated Hospital of Nanjing Medical University (Nanjing, China).

### Primer Design

We found type strains of the seven LAB genera (*Lactobacillus*, *Streptococcus*, *Weissella*, *Lactococcus*, *Pediococcus*, *Enterococcus*, and *Leuconostoc*) of interest in LPSN^1^, downloaded their corresponding 16S rRNA gene sequence from NCBI^2^, and combined them in a LABSEQ.fasta file. Consistent sequences were obtained by comparing the 16S rRNA gene sequences from all strains from which potential single-stranded primers could be designed and modified using degenerate bases. We assembled primer pairs with product sizes of >400 bp. To ensure that the primer matched all the target LAB species, all potential primers were compared with the sequences in the LABSEQ.fasta file using the “search” function in Mega 6.0 software.

### Characterisation of LAB in Koumiss and Fecal Samples

Koumiss is a milk product fermented by LAB and yeast; LAB are abundant in koumiss (Danova et al., 2005; Yao et al., 2017). The koumiss sample we used was made using traditional fermentation methods by a family in Xilinhot, Inner Mongolia. Three fecal samples, designated Sample 1, Sample 2, and Sample 3, were used during the evaluation procedure of selected primers by single-molecule real-time (SMRT) sequencing. In this study, fecal Samples 1 and 2 were from a healthy individual who had been screened and accepted as a donor for fecal microbiota transplantations by China fmtBank (Zhang et al., 2018), Sample 1 was the original fecal material from the healthy donor and Sample 2 was the purified suspension of microbiota from Sample 1. Microbiota from Sample 1 was processed in an automatic purification system (GenFMTer, FMT Medical, China) as described previously by Cui et al. (2015). Sample 3 was from a donor with Irritable Bowel Syndrome (IBS) who had not received antibiotics in the previous 3 months.

### DNA Extraction and PCR Amplification

Bacterial DNA from representative reference strains from the culture collection (**Table 1**) was extracted from 2 ml of the culture medium using TIANamp Bacteria DNA Kit (TIANGEN, Beijing) following the manufacturer’s instructions. Bacterial DNA from 5 ml of naturally fermented koumiss or 0.5 g of feces was extracted using the QIAamp Stool Mini Kit (QIAGEN, Germany) following the manufacturer’s instructions. All DNA was stored at -20°C prior to evaluation.

**Table 1:** Strains used in this study.

Genus	Type species	Reference number
*Enterococcus*	*Enterococcus asini*	DSM 11492^T^
	*Enterococcus faecium*	ATCC 19434^T^
	*Enterococcus italicus*	DSM 15952^T^
*Lactobacillus*	*Lactobacillus casei*	ATCC 334^T^
	*Lactobacillus salivarius*	DSM 20555^T^
	*Lactobacillus helveticus*	DSM 20075^T^
*Pediococcus*	*Pediococcus acidilactici*	JCM 8791^T^
	*Pediococcus pentosaceus*	DSM 20336^T^
*Streptococcus*	*Streptococcus thermophilus*	NM-81-2
*Weissella*	*Weissella beninensis*	DSM 22752^T^
	*Weissella confusa*	IMAU10245
	*Weissella kandleri*	DSM 20593^T^
*Leuconostoc*	*Leuconostoc pseudomesenteroides*	DSM 20193^T^
*Lactococcus*	*Lactococcus plantarum*	DSM 20686^T^
Non-LAB	*Shigella castellani*	ATCC 29903^T^
	*Escherichia coli*	ATCC 11775^T^


*Type strains are indicated with a superscript T.*

PCR for primer specificity testing was performed in a final volume of 50 μL containing 5 μL 10× PCR buffer, 4 μL dNTP mix (2.5 mmol/L), 1.2 μL forward primer (10 μmol/L), 1.2 μL reverse primer (10 μmol/L), 0.5 μL Taq DNA polymerase (5 U/μL), 2 μL extracted DNA (∼20 ng/μL), ddH_2_O to a total volume of 50 μL. The reagents were purchased from Dalian Baosheng Biological Engineering Company (Dalian, China).

PCR for SMRT sequencing was performed in a final volume of 50 μL containing 25 μL 2 × KAPA HiFi HotStart ReadyMix, 1.2 μL forward primer (10 μM), 1.2 μL reverse primer (10 μM), 1 μL extract DNA (20 ng/μL), ddH2O to a total volume of 50 μL. The reagents were purchased from Pacific Biosciences (Menlo Park, CA, United States). PCR amplifications were achieved using the following program: pre-denaturation at 95°C for 10 min, then 30 cycles of denaturation at 95°C for 30 s, annealing for 1 min, and extension at 72°C for 1.5 min. The last cycle was followed by a 7 min extension at 72°C.

### Primer Specificity Evaluation

From the primers that were created we selected those with product sizes of >400 bp. We used *Lactobacillus salivarius* and *Lactobacillus helveticus* as DNA templates to screen for primers with amplification products that appeared as single clear bands on the gel and then determined their annealing temperatures. Subsequently, fourteen LAB strains and two non-LAB strains that were closely related to the target genera were used to evaluate the specificity of the selected primers based on PCR amplicons and agarose gel electrophoresis. All strains were provided by the Lactic Acid Bacteria Culture Collection of Inner Mongolia Agricultural University (Hohhot, China). Detailed information of the strains is presented in **Table 1**.

### Primary Evaluation of Primers Using SMRT Sequencing

Using SMRT sequencing we sequenced PCR amplicons from bacterial DNA extracted from the koumiss sample (which is known to have a high abundance of LAB) with LAB primers L1, L2, L5, L6, and the universal bacterial primer (27f/1492r; 5′-AGAGTTTGATCCTGGCTCAG-3′/5′-CTACGGCTACCTTGTTACGA-3′). Initially, the sequence reads were randomly extracted based on the sample with the fewest sequences. Sequences were then assigned to OTUs using UCLUST (Lozupone and Knight, 2005; Edgar, 2010) after selection of representative sequences with a 100% pairwise identity threshold; these were then classified taxonomically using Greengenes (DeSantis et al., 2006), RDP (Cole et al., 2007) and Silva (Pruesse et al., 2007) databases. Finally, a heatmap was drawn using the “pheatmap” package^3^ in R to determine presence or absence of target bacterial species in the koumiss sample.

To further investigate the efficiency of the primer candidates and choose the best LAB group-specific sequence primer, we amplified and sequenced bacterial DNA extracted from the three fecal samples (Samples 1, 2, and 3 which are presumed to have a low relative abundance of LAB) with the universal bacterial primer (27f/1492r) and LAB-specific primers L5 and L6. The analytical process used on the sequencing data was the same as that used on the data from koumiss. The alpha diversity was evaluated by the observed OTUs and Shannon diversity index. A histogram of the Shannon diversity index results was created using Microsoft Excel and significant differences were calculated by Mann-Whitney or Kruskal-Wallis test in SPSS ver. 20 software (IBM Corp., Armonk, NY, United States).

### Building a Phylogenetic Tree Based on the Optimal Primer Created

In order to ensure the ability to infer phylogeny amongst closely related bacterial taxa from amplicons that had been amplified with the LAB-specific primer L5, we obtained alignments between the forward and reverse primer of L5 in the LABSEQ.fasta file and deleted mismatching alignments at the 3′ end of primer L5. A phylogenetic tree (designated as the L5-Tree) was created on the 3′ end of primer L5 using TreeBeST software (V1.9.2), and Visual trees were developed using FigTree software (V1.4.3). The distances between pairs of strain sequences were calculated using Mega 6.0 software. At the same time, corresponding 16S rRNA gene sequences in the LABSEQ.fasta file were selected as controls, on which a phylogenetic tree (designated as the 16S-Tree) was created and the distances between pairs of strain sequences were calculated.

### Nucleotide Sequence Accession Numbers

Raw sequence data are publicly available online through MG-RAST project number mgp86134^4^.

## Results

### Primer Design

443 type strains belonging to seven LAB genera (*Lactobacillus*, *Streptococcus*, *Weissella*, *Lactococcus*, *Pediococcus*, *Enterococcus*, and *Leuconostoc*) were found, and their corresponding 16S rRNA gene sequences were downloaded in the LABSEQ.fasta file. We obtained five consensus primer-binding sites by aligning the 16S rRNA gene sequences in the LABSEQ.fasta file, from which 16 single-stranded candidate LAB primers (11 forward primers and 5 reverse primers) were designed manually. A total of 31 primer pairs were assembled with product sizes of >400 bp. The primers were between 17 and 24 bp long with no more than two degenerate bases.

### Primer Specificity Analysis Using Agarose Gel Electrophoresis

Of the 31 pairs of primers, six primer pairs with product sizes of >400 bp and single clear bands on the gel were selected using PCR amplification with agarose gel electrophoresis and taking *L. salivarius* and *L. helveticus* as DNA templates. The optimum annealing temperature (T_m_) was determined (**Table 2**). From this, 14 strains belonging to seven LAB genera and two non-LAB strains (**Table 1**) were used to test the specificity of the six primers using PCR amplification and agarose gel electrophoresis. The results showed that primers L3 (∼600 bp) and L4 (∼400 bp) amplified both LAB and non-LAB strains, while primers L1 (∼1000 bp), L2 (∼1000 bp), L5 (∼750 bp), and L6 (∼1000 bp) only amplified the 14 LAB strains, showing increased specificity for LAB species (**Supplementary Figure S1**). Thus L1, L2, L5, and L6 were selected for further study.

**Table 2:** Information about the selected lactic acid bacteria primers.

Primers	Position	Sequence (5′–3′)	Tm	Product size
L1	13f	TGGCTCAGGAYGAACGCYG	60°C	∼1000 bp
	957r	TCGAATTAAACCACATGCTCCA		
L2	13f	TGGCTCAGGAYGAACGC	60°C	∼1000 bp
	957r	GAGGCWGCAGTAGGGAATC		
L3	361f	TCCGGAWTTATTGGGCGTAAAG	60°C	∼600 bp
	957r	TCGAATTAAACCACATGCTCCA		
L4	567f	TCGAATTAAACCACATGCTCCA	60°C	∼400 bp
	957r	TCGAATTAAACCACATGCTCCA		
L5	15f	GCTCAGGAYGAACGCYGG	60°C	∼750 bp
	687r	CACCGCTACACATGRADTTC		
L6	19f	AGGAYGAACGCYGGCGGCGTGCC	60°C	∼1000 bp
	957r	TCGAATTAAACCACATGCTCCA		


*The relative position for the six identified LAB-specific primers was based on *Lactobacillus salivarius* 16S rRNA gene sequences (Accession number: AF089108.2).*

### Primary Evaluation of Primers Using SMRT Sequencing

**FIGURE 1:**
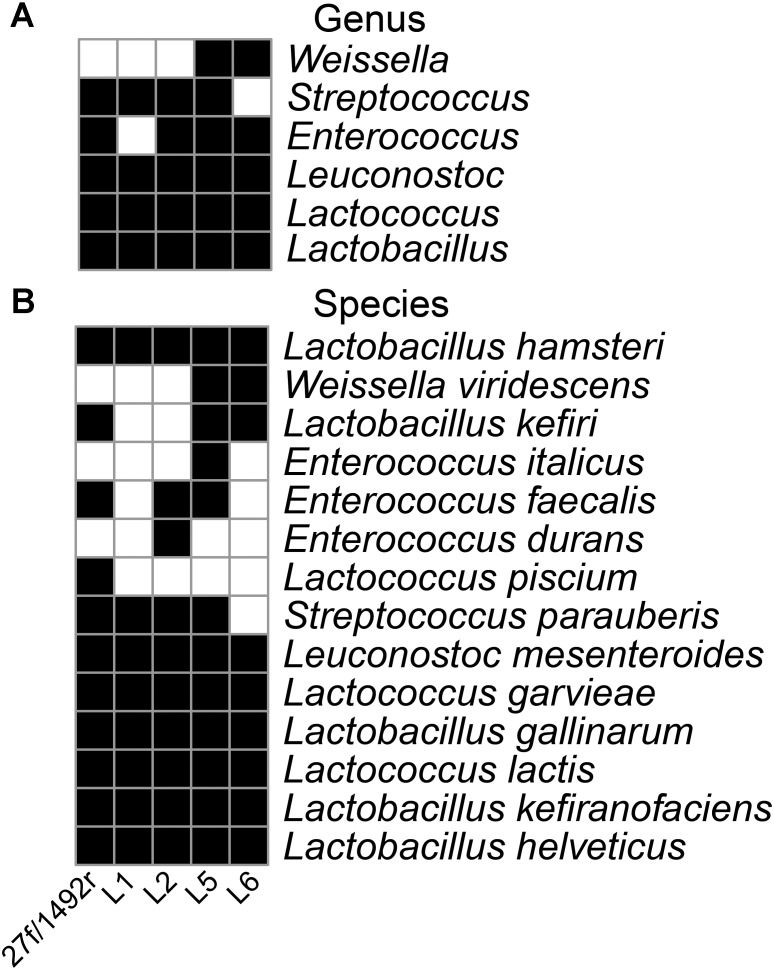
Diversity of LAB in a koumiss sample identified using various primers. A koumiss sample with a high relative abundance of LAB was evaluated using SMRT sequencing of amplicons. A heatmap was created according to the presence (black squares) and absence (white squares) of the target bacteria at the genus **(A)** and species **(B)** level.

The 27f/1492r, L1, L2, L5, and L6 amplicons from the koumiss sample generated 4458, 655, 682, 1293, and 1809 sequence reads with target sequence read proportions of 73, 94, 81, 93, and 97%, respectively (**Table 3**). The diversities of the L1, L2, L5, and L6 amplicons from the koumiss sample were similar to that of 27f/1492r at the genus level after standardizing for sequence quantity. However, *Weissella* was detected in the L5 and L6 amplicons but not in the 27f/1492r amplicon. At the species level, ten target LAB species were detected in the 27f/1492r amplicon, while eight, ten, twelve, and nine target LAB species were detected in the L1, L2, L5, and L6 amplicons, respectively (**Figure 1**). Interestingly, *Lactococcus piscium* was detected in the 27f/1492r amplicon from the koumiss sample, but not in the four LAB primer amplicons. Overall, the target sequence read proportions for L5 and L6 were higher and could amplify more target LAB than primers 27f/1492r, L1 and L2. Therefore, we considered L5 and L6 as candidate LAB primers with the greatest potential for further evaluation.

**Table 3:** Sequencing reads from samples amplified by various primers.

Samples	Koumiss					Sample 1			Sample 2			Sample 3		
				
Primers	27f/1492r	L1	L2	L5	L6	27f/1492r	L5	L6	27f/1492r	L5	L6	27f/1492r	L5	L6
Sequencing total reads	4458	655	682	1293	1809	6880	2551	6221	7696	3405	4463	3844	3222	3431
Target sequencing reads	3262	613	554	1199	1754	56	356	345	60	432	279	90	327	299
Proportion (%)	73	94	81	93	97	0.8	14	6	0.8	13	6	2	10	9


### Further Evaluation of the Primer Candidates Using Fecal Samples

Three fecal samples (Sample 1, Sample 2, and Sample 3) were amplified with 27f/1492r, L5, L6 and sequenced using SMRT technology. For Sample 1 and Sample 2, the proportions of target reads in 27f/1492r, L5 and L6 amplification products were as follows: 0.8, 14, 6% (Sample 1) and 0.8, 12.68, 6.27% (Sample 2). For Sample 3, the proportion of target reads in amplification products of 27f/1492r, L5 and L6 were 2, 10, and 9%, respectively (**Table 3**).

**FIGURE 2:**
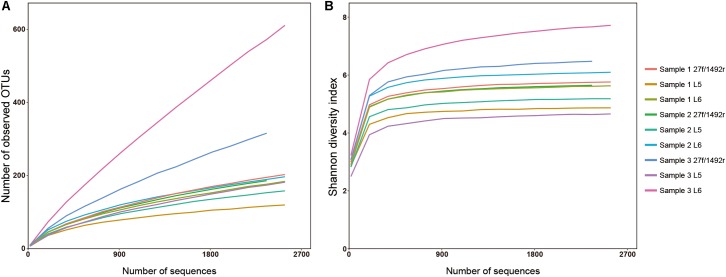
**(A)** Rarefaction and **(B)** Shannon diversity of DNA sequences from three fecal samples amplified using the universal bacterial primer 27f and candidate LAB-specific primers. Sample 1, Sample 2, and Sample 3, were amplified using the universal bacteria primer 27f and LAB-specific primers L5 and L6 and sequenced using SMRT sequencing.

**FIGURE 3:**
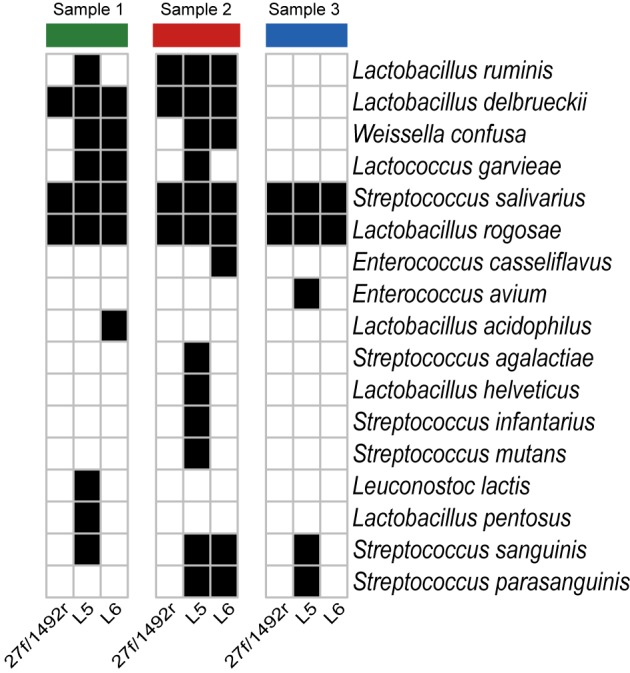
Species of bacteria identified in fecal samples using candidate primers after standardizing the sequence counts across samples. Three fecal DNA samples were amplified using the universal bacteria primer 27f and LAB-specific primers L5 and L6 and then sequenced using SMRT sequencing. A heatmap was created according to the presence (black squares) and absence (white squares) of the target LAB bacteria at the species level after standardizing sequence counts across samples.

A total of 2551 sequences were randomly selected from each sample to standardize sequence quantity from different samples. Shannon-Wiener diversity curves showed that the sequence depth was adequate for all samples. Although new phylogroups are likely to be discovered along with an increase in sequencing depth, the data presented here show that most of the LAB diversity had been captured (**Figure 2**). We found that, at the species level, three, nine and six LAB species were detected in Sample 1; four, twelve and eight species were detected in Sample 2; and two, five and two species were detected in Sample 3 when amplified by 27f/1492r, L5 and L6 primers, respectively (**Figure 3**). The relative abundances of LAB are listed in **Table 4**. Not only did primers L5 and L6 detect all target LAB species found in the 27f/1492r amplicons, but also more target LAB species that were not detected in the 27f/1492r amplicons; of these primer L5 had the greatest amplification diversity.

**Table 4:** The relative proportion of LAB species identified from samples of feces by group-specific primers (%).

Taxonomy/primers	Sample 1			Sample 2			Sample 3		
			
	27f/1492r	L5	L6	27f/1492r	L5	L6	27f/1492r	L5	L6
*Enterococcus avium*	0	0	0	0	0	0	0	0.07	0
*Enterococcus casseliflavus*	0	0	0	0	0	0.07	0	0	0
*Lactobacillus acidophilus*	0	0	0.07	0	0	0	0	0	0
*Lactobacillus delbrueckii*	0.07	0.26	0.07	0.08	0.19	0.07	0	0	0
*Lactobacillus helveticus*	0	0	0	0	0.06	0	0	0	0
*Lactobacillus pentosus*	0	0.06	0	0	0	0	0	0	0
*Lactobacillus rogosae*	0.35	0.26	2.36	0.15	0.06	1.77	1.78	0.7	3.57
*Lactobacillus ruminis*	0	0.13	0	0.08	0.88	0.2	0	0	0
*Lactococcus garvieae*	0	0.06	0.07	0	0.06	0	0	0	0
*Leuconostoc lactis*	0	0.13	0	0	0	0	0	0	0
*Streptococcus agalactiae*	0	0	0	0	0.06	0	0	0	0
*Streptococcus infantarius*	0	0	0	0	0.06	0	0	0	0
*Streptococcus mutans*	0	0	0	0	0.06	0	0	0	0
*Streptococcus parasanguinis*	0	0	0	0	0.06	0.13	0	0.07	0
*Streptococcus salivarius*	0.35	10.89	1.64	0.3	9.78	3.02	0.07	4.81	0.14
*Streptococcus sanguinis*	0	0.06	0	0	0.13	0.07	0	0.07	0
*Weissella confusa*	0	0.58	0.33	0	0.19	0.07	0	0	0


### Evaluation of Target LAB Diversity as Amplified by Candidate LAB-Specific Primers

**FIGURE 4:**
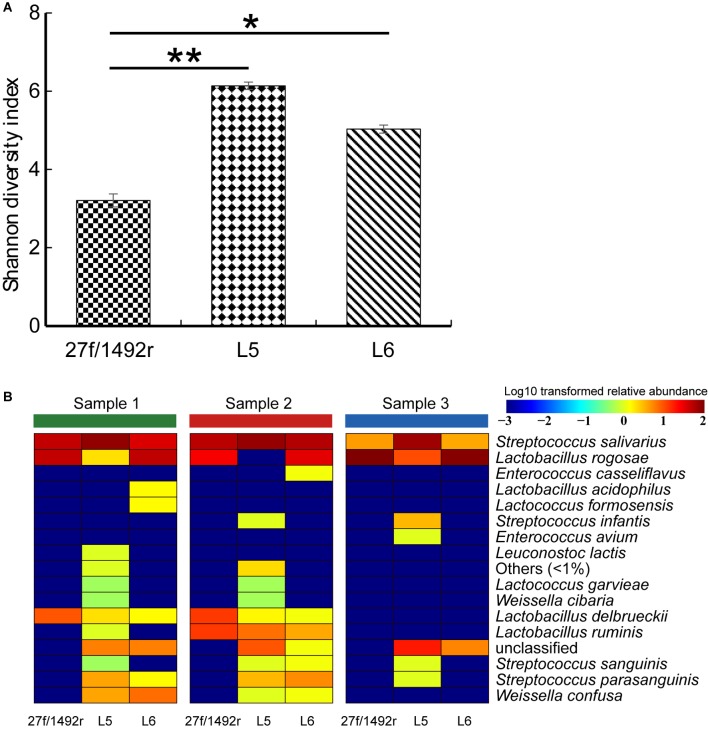
**(A)** Shannon diversity index and **(B)** diversity of target LAB OTUs in three fecal DNA samples amplified using candidate LAB-specific primers. **(A)** All target LAB OTUs were extracted from the sequence data, the Shannon diversity index was calculated, a histogram was created and significant differences were tested. **(B)** All target LAB OTUs were annotated (QIIME ver. 7.0) and a heatmap was created according to the presence and relative abundance of the target bacteria at the species level. Significant difference at ^∗^*p* < 0.05 and ^∗∗^*p* < 0.01.

The diversity of the target LAB was significantly higher (*p* < 0.05) in L5 and L6 amplicons than in 27f/1492r amplicons (**Figure 4A**). We also created a heatmap according to the relative abundance of the target LAB extracted from the total sequence at the species level (**Figure 4B**). More species were detected in the same fecal sample when amplified using L5 than when using the other two primers (**Supplementary Figure S1**). Furthermore, some target LAB species with low relative abundance were detected in the L5 amplicons but not in the 27f/1492r and L6 amplicons, indicating that primer L5 was more efficient in detecting target LAB species with low relative abundances in complex samples.

### Phylogenetic Tree Analysis

**FIGURE 5:**
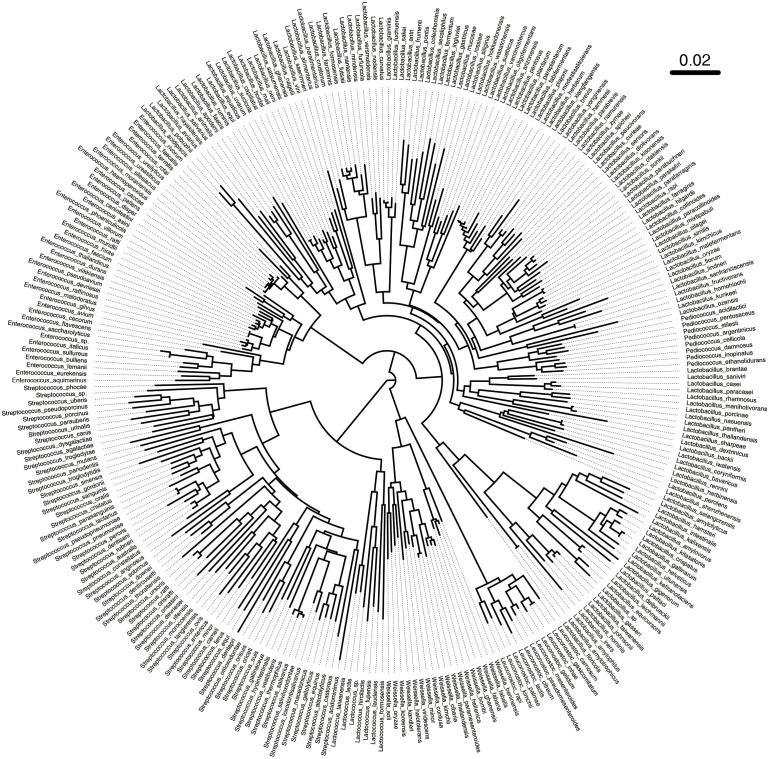
Phylogenetic tree based on sequences between the forward and reverse primer L5. A total of 347 sequences between the forward and reverse primer of L5 were obtained in LABSEQ. A phylogenetic tree was constructed based on these sequences using TreeBeST software (V1.9.2). Visual trees were then developed using FigTree software (V1.4.3).

Based on the above results, we found that primer L5 had the highest amplification efficiency for target LAB. In view of this, we further studied the discriminatory ability of L5 primers for different LAB strains. We obtained 347 alignments between the forward and reverse primer of L5 in LABSEQ.fasta, built a phylogenetic tree based on this, and calculated the distances between pairs of sequences. The L5-tree showed that seven genera of LAB could be distinguished from each other, while partial species in the same genera clustered together, especially *Enterococcus* and *Lactobacillus* (**Figure 5**). For example, *Enterococcus caccae* clustered together with: *Enterococcus haemoperoxidus*, *Enterococcus moraviensis*, *Enterococcus rotai*, *Enterococcus silesiacus*, *Enterococcus ureasiticus*, *Enterococcus ureilyticus*, and *Enterococcus termitis*. *Lactobacillus frutivorans* clustered with *Lactobacillus homohiochii*, *Lactobacillus amylophilus*, and *Lactobacillus amylolyticus*. *Lactobacillus uvarum* clustered with *Lactobacillus aquaticus*, *Lactobacillus arizonensis*, and *Lactobacillus pentosus*. *Leuconostoc gasicomitatum* and *Leuconostoc inhae* also clustered together (**Figure 5**). We found similar results in the phylogenetic tree based on the full length of 16S sequences (**Supplementary Figure S2**). Moreover, with 98% identity, only about 0.7% of all paired strains in L5-tree were difficult to distinguish from each other as different species. In the 16S-tree about 3.27% of all paired strains were difficult to distinguish from each other as different species.

## Discussion

LAB abundance and diversity influences human health and the production of many fermented foods. However, the relative abundance of LAB in some complex biological samples, such as feces, can be low and often neglected in research studies (Turroni et al., 2012; Nakayama et al., 2015; Gough et al., 2016; Zheng et al., 2016). This hinders studies on the population structure, distribution and diversity of LAB in samples, and the correlation between LAB and other members of the microbiota.

Previously designed primers are only able amplify up to four different LAB genera and are mostly used for quantification of LAB, especially *Lactobacillus* (Heilig et al., 2002; Nielsen et al., 2003; Byun et al., 2004; Cagno et al., 2009). One LAB primer pair has been reported to amplify *Lactobacillus*, *Leuconostoc*, *Pediococcus* and *Weissella* (Walter et al., 2001) and another to amplify *Lactobacillus*, *Lactococcus*, *Leuconostoc*, and *Pediococcus* (Lopez et al., 2003) with product sizes of about 340 and 400 bp, respectively. Such short product sizes can only be used to analyze the composition of some LAB populations at the genus level. Apart from these no LAB primers have been designed with product sizes that can be used to identify the diversity of LAB at the species level. In this study, the length of LAB primer L5 is about 750 bp, and it was able to amplify *Lactobacillus*, *Lactococcus*, *Leuconostoc*, *Enterococcus*, *Weissella*, and *Streptococcus* at the same time. Phylogenetic analysis showed that primer L5 was also capable of determining the species composition of those seven genera. We also found that primer L5 significantly increased the proportion of LAB in the sequencing results. This could significantly reduce the sequencing cost of LAB studies in complex samples.

In this study, no *Pediococcus* species were detected in the L5 amplicons using SMRT sequencing, whereas *Pediococcus acidilactici* (JCM 8791) and *Pediococcus pentosaceus* (DSM 20336) were amplified by L5 in agarose gel electrophoresis during primer screening. *Pediococcus* is comprised of 12 species^1^, and different strains of *Pediococcus* have been isolated from feces (Millette et al., 2008; Mathys et al., 2009; Rodriguez-Palacios et al., 2009), fermented sausage (Albano et al., 2007; Cosansu et al., 2007), sauerkraut (Zhi-Jiang et al., 2006), and the rumen of sheep (Cobos et al., 2011). The most likely reason why *Pediococcus* was not detected in this study is because the abundance of *Pediococcus* species in the selected samples was too low, and the current sequencing depth does not cover these bacteria.

We evaluated the ability to use primer L5 to distinguish between different LAB species using a phylogenetic tree constructed based on the sequences between the forward and reverse primer of L5. We compared this with the phylogenetic tree based on the full-length of the 16S. With the exception of partial species in the same genera primer L5 differentiated between most target LAB species very well. These species are difficult to distinguish, even based on the full-length 16S phylogenetic tree, because 16S rRNA gene sequences are highly conserved between different species of bacteria (Coenye and Vandamme, 2010). This has been reported previously (Turroni et al., 2009; Milani et al., 2014). Taken together these findings demonstrate how robust the use of sequences between the forward and reverse primer L5 to infer phylogenetic relatedness amongst species of LAB; primer L5 was the more suitable for compositional assessment of LAB populations, at the species level, in complex ecosystems.

LAB are a complex polyphyletic group that are always present in the intestine and various fermented foods. LAB-specific primer L5 designed in this study was able to distinguish between LAB in a complex ecosystem. This study provides a foundation for further research on the diversity of LAB in complex samples. The methods used in this study may be a useful reference for other primer design studies.

## Author Contributions

HZ and ZS designed the experiments. XB and XG performed the experiments. QH and WL analyzed the data. FZ provided fecal samples from donors. XB and QH wrote the manuscript. All authors read and approved the final manuscript.

## Conflict of Interest Statement

The authors declare that the research was conducted in the absence of any commercial or financial relationships that could be construed as a potential conflict of interest.

## Footnotes

1 http://www.bacterio.net/

2 http://www.ncbi.nlm.nih.gov/guide/

3 https://cran.r-project.org/web/packages/pheatmap/index.html

4 http://www.mg-rast.org/linkin.cgi?project=mgp86134

## Supplementary Material

FIGURE S1 Target LAB diversity in fecal samples amplified using candidate primers.

FIGURE S2 Phylogenetic tree based on 16S rRNA sequences. A total of 347 corresponding 16S rRNA sequences were obtained in LABSEQ. A phylogenetic tree was constructed based on these using TreeBeST software (V1.9.2). Visual trees were then developed using FigTree software (V1.4.3).
