# Supplementary figures and images for: Design of Primers for Evaluation of Lactic Acid Bacteria Populations in Complex Biological Samples
Source: Front Microbiol. 2018 Aug 31;9:2045. doi: 10.3389/fmicb.2018.02045 (PMC6127287; doi:10.3389/fmicb.2018.02045)

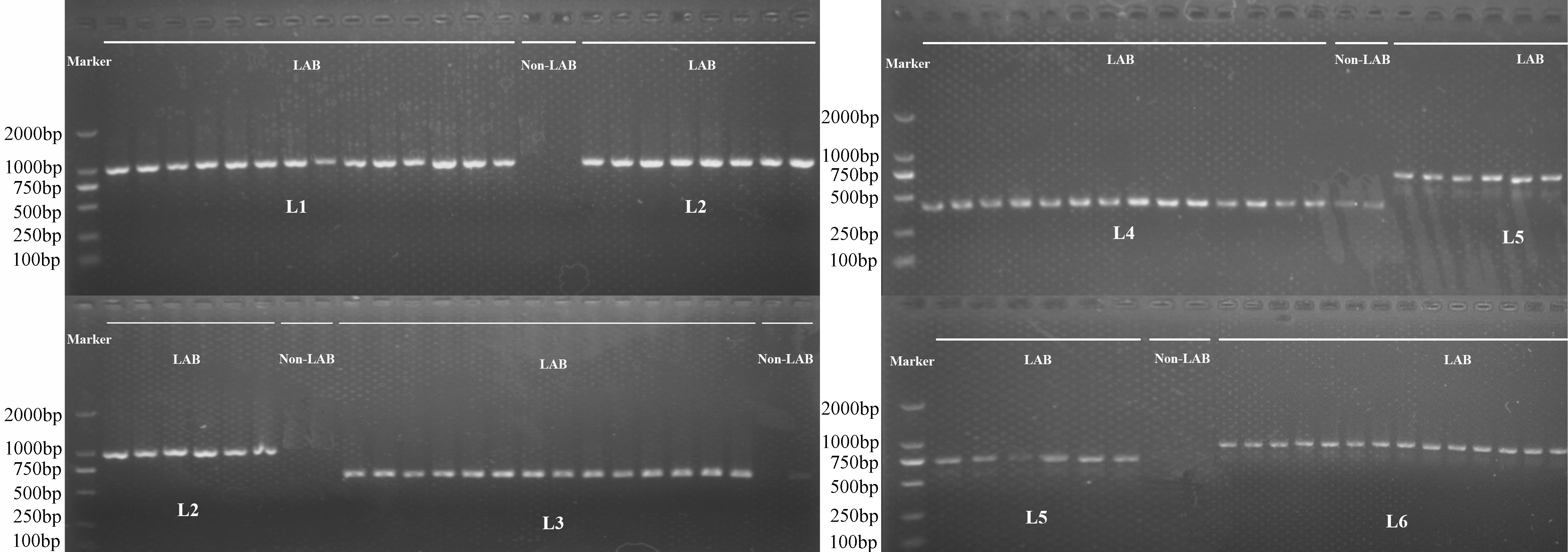

Supplement: FIGURE S1 — Target LAB diversity in fecal samples amplified using candidate primers. [file Image_1.TIF]

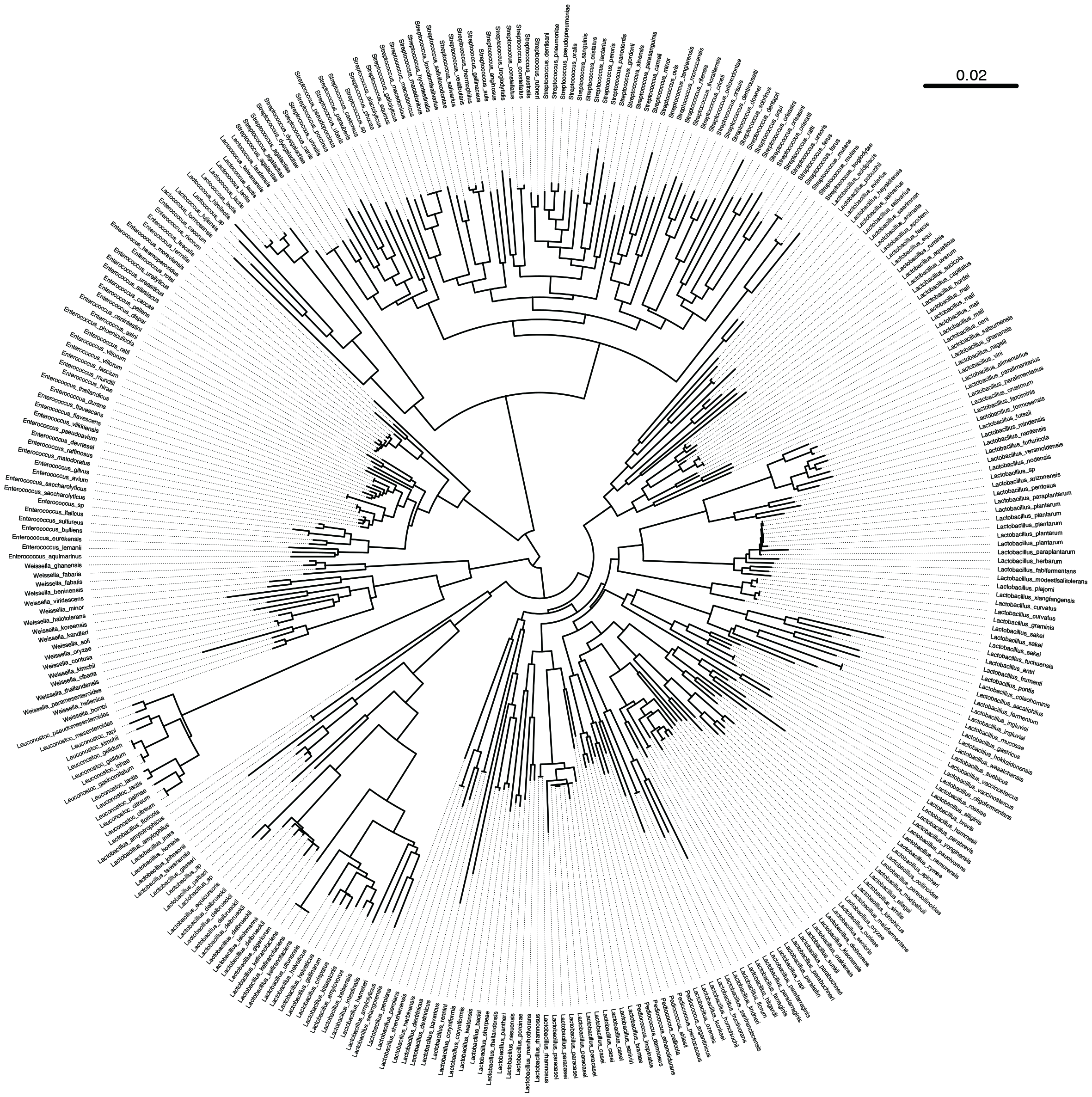

Supplement: FIGURE S2 — Phylogenetic tree based on 16S rRNA sequences. A total of 347 corresponding 16S rRNA sequences were obtained in LABSEQ. A phylogenetic tree was constructed based on these using TreeBeST software (V1.9.2). Visual trees were then developed using FigTree software (V1.4.3). [file Image_2.TIF]
